# Clinical parameters to guide decision-making in elderly metastatic colorectal cancer patients treated with intensive cytotoxic and anti-angiogenic therapy

**DOI:** 10.18632/oncotarget.14333

**Published:** 2016-12-28

**Authors:** Gemma Bruera, Antonio Russo, Antonio Galvano, Sergio Rizzo, Enrico Ricevuto

**Affiliations:** ^1^ Oncology Territorial Care, S. Salvatore Hospital, Oncology Network ASL1 Abruzzo, University of L’Aquila, L’Aquila, Italy; ^2^ Department of Biotechnological and Applied Clinical Sciences, University of L’Aquila, L’Aquila, Italy; ^3^ Medical Oncology, Department of Surgical, Oncological and Stomatological Sciences, University of Palermo, Palermo, Italy

**Keywords:** elderly, intensive treatment, metastatic colorectal cancer, triplet chemotherapy plus bevacizumab, unfit

## Abstract

**Introduction:**

Bevacizumab addiction to triplet chemotherapy, according to FIr-B/FOx schedule, as first-line treatment in young-elderly metastatic colorectal CANCER (MCRC) patients may be more effective. Tailored treatments show worse clinical outcome in unfit patients.

**Methods:**

Elderly patients were clinically evaluated according to age and comorbidity (Cumulative Illness Rating Scale) to select FIr-B/FOx regimen in fit or tailored treatments in unfit elderly. Limiting toxicity syndromes (LTS) were evaluated.

**Results:**

At 17 months follow-up, in 28 young-elderly patients treated with first line FIr-B/FOx: objective response rate (ORR) 79%, progression-free survival (PFS) 11 months, overall survival (OS) 21 months. Clinical outcome was not significantly different according to *KRAS* genotype. G3-4 toxicities were diarrhea 21%, mucositis 11%, neutropenia 11%. LTS were 46%, significantly more multiple than single site. At 8 months follow-up, in 37 unfit patients: ORR 37%, PFS 7 months, OS 13 months. PFS was significantly different in *KRAS* wild-type compared to mutant patients, while not OS. PFS and OS were significantly worse in *KRAS* c.35 G > A compared to wild-type and/or other mutant.

**Conclusions:**

Careful decision-making process including evaluation of patient's fitness, and individual safety should be included to select FIr-B/FOx intensive first line regimen in young-elderly MCRC patients. *KRAS*, and specifically c.35 G > A mutant genotype, may significantly affect clinical outcome in patients unfit for FIr-B/FOx.

## INTRODUCTION

Different treatment options and lines of medical treatment in metastatic colorectal cancer (MCRC) patients are currently tailored according to fitness (age, performance status (PS), comorbidities), metastatic extension (liver-limited (L-L) or other/multiple metastatic (O/MM)), and *RAS* genotype [1–7]. First line regimens, consisting of triplet chemotherapy, or bevacizumab (BEV) or cetuximab or panitumumab in addiction to doublet chemotherapeutic drugs, showed overlapping activity and clinical outcome in phase III trials: objective response rate (ORR) 39%-68%, progression-free survival (PFS) 7.2-10.6 months, and overall survival (OS) 19.9-26.1 months [3, 5, 7, 8, 1]. In ‘fit’ patients, these treatment options in first-line setting, integrated with secondary liver metastasectomies, significantly increased clinical outcome over doublet chemotherapy. More intensive triplet chemotherapy plus BEV can further achieve ORR 82%, liver metastasectomies 26%, PFS 12 months, OS 28 months [2–4, 9, 10, 6]. In L-L disease, metastasectomies were 54% and clinical outcome was significantly improved, particularly in *KRAS* wild-type patients [4, 6]. The selection among intensive and more effective or tailored first-line medical treatment, with a proper balance between expected efficacy and safety, also according to prognostic parameters (extension of metastatic disease in terms of involved sites (liver-limited, other/multiple metastatic sites), *KRAS* genotype) represents a major challenge in clinical management of MCRC patients.

MCRC patients are prevalently elderly but often under-treated in clinical practice, and usually underrepresented in clinical trials. They require a decision-making process combining the evaluation of fitness, according to co-morbidity, functional, and nutritional status [11], and selection of proper medical treatment with increasing effectiveness weighed by non-limiting toxicity and maintained quality of life (QoL). Clinical characteristics limiting fitness for intensive medical treatments are elderly status (>=75 years), PS ≥2, and/or comorbidities. Retrospective studies showed similar safety and efficacy in fit elderly compared to younger patients [12–14]. Elderly patients benefited from 5-fluorouracil (5-FU) [15–17], irinotecan (CPT-11)-containing chemotherapy [18, 19], FOLFOX [20] as younger patients [20–22]. In fit patients ≥70 years, 5-FU reported ORR 23.9%, PFS 5.5 months, OS 10.8 months [15]. The same clinical benefit and tolerability were reported from CPT-11-containing chemotherapy [18]; age did not represented an independent prognostic factor correlated to OS [19]. FOLFOX conferred a significantly improved relative benefit independently from age [20]. In old-elderly and younger patients treated with FOLFOX, (OPTIMOX1 trial), activity and efficacy data were comparable: ORR 59%, median PFS 9.0 months, and median OS 20.7 months [21]. In the FOCUS2 trial, specifically designed to prospectively evaluate first line reduced-dose (80%) of 5-FU or capecitabine added or not to OXP in old-elderly and/or frail patients, OXP addition did correlated with a benefit in terms of OS and above all PFS (5.8 vs. 4.5 months, p 0.07), the primary endpoint of the study, but with a significantly improved ORR (35% vs 13) [22]; combination therapy did not significantly modify safety profile, but showed worse QoL. Treatment efficacy was consistent across subgroups, including age, when BEV was combined with CPT-11-based therapy [23]. In fit elderly patients, BEV addition to 5-FU-containing chemotherapy conferred significantly longer PFS (9.2 months) and OS (17.4-19.3 months) [24, 25]. In BRiTE and BEAT trials, which are two large trials that evaluate the association between doublet chemotherapy plus bevacizumab, PFS was not different (about 10.0 months); median OS decreased with age [25, 26]. The use of bevacizumab plus chemotherapy in this setting of patients was also investigated in other two phase II studies. The results of AXELOX [43] and BECOX [44] trials suggested that the combination of bevacizumab to doublet chemotherapy could be an acceptable first line option for older mCRC patients.

No impact on PFS and OS was observed by age and/or comorbidities in patients treated with FOLFOX or FOLFIRI added or not to cetuximab [27]. Addition of panitumumab to FOLFOX showed no clear benefit in PFS in elderly patients, and with PS 2 [29]. PS 1 compared to PS 2 significantly modified clinical outcome, regardless of medical treatment administered, as showed in a meta-analysis: ORR 43.8% vs 32%, PFS 7.6 vs 4.9 months, OS 17.3 and 8.5 months, respectively [30]. In the phase III randomized trial comparing FOLFOXIRI with FOLFIRI, age was not a significant parameter affecting activity and efficacy; elderly patients showed median OS 19.9 and 16.9 months, respectively [31, 32]. Activity was significantly lower in older patients enrolled in the FOLFOXIRI arm [32]; no differences were reported in PFS and OS. Patients who underwent metastasectomies didi not showed significantly more morbidity and/or mortality, independently from age. Patients with PS 2 showed significantly worse clinical outcome in both FOLFIRI and FOLFOXIRI arm [31, 32].

Recent retrospective analyses reported by our group, evaluating clinical outcome and safety of first-line FIr-B/FOx intensive regimen in fit young-elderly MCRC patients [2, 33], or tailored medical treatments in patients unfit, due to age and/or comorbidities [7], point out the need to integrate the evaluation of patient's fitness and selection of first line medical treatment in a proper decision-making process in elderly MCRC patients.

## RESULTS

### Effectiveness and safety of triplet chemotherapy-based intensive regimens in elderly MCRC patients

#### Intensive triplet chemotherapy

HORG-FOLFOXIRI schedule proposed by Soug-lakos *et al*. [32], characterized by reduced projected DI (pDI) of CPT-11 (65 mg/m^2^/w), OXP (32.5 mg/m^2^/w), associated to bolus plus continuous infusion 5-FU (pDI 1000 mg/m^2^/w; received dose intensity (rDI) 880 mg/m^2^/w (88%)) failed to confirm significantly improved clinical efficacy compared to FOLFIRI in unresectable MCRC patients [3, 32]. Different patients’ selection, DI of drugs, and/or 5-FU administration modality may justify these results [5]. Elderly patients were prevalently enrolled (56%, median age 66 years) and PS was poorer; elderly patients >70 years and also >75 years with an ECOG PS 1 or higher were enrolled. Elderly patients were not evaluated for comorbidity and functional status. The study reported a significantly higher incidence of toxicity in older patients and those with PS 2; PS 2 patients showed a significantly lower median OS and PFS, in both arms [31, 5].

In an unplanned subgroup evaluation of the phase III study, prognostic relevance of elderly status in both FOLFIRI and FOLFOXIRI treated patients was evaluated [32], and clinical outcome was not significantly different. In elderly patients treated with FOLFIRI, compared with younger, no significantly different activity and efficacy data were reported: ORR 31.7%; time to tumor progression (TTP) 6.2 months; median OS 17.8 months (Table 1). In patients treated with FOLFOXIRI, ORR was significantly lower in elderly compared to younger patients, 32% vs 52% (p = 0.03); however, TTP (8.5 vs. 9.6 months, respectively; p = 0.54) and median OS (19.9 vs. 23 months, respectively; p = 0.47) were not significantly different.

**Table 1:** Activity and efficacy in elderly MCRC patients

	FIr-B/FOx	FOLFOXIRI	FOLFIRI
**Enrolled patients, No**.	28	75	82
**Objective Response, %**	79	32	34
**Median progression-free survival, months**	11	8.5	6.2
**Median overall survival, months**	21	19.9	17.8

HORG/FOLFOXIRI intensive regimen showed a worse safety profile compared to FOLFIRI, with significantly more dose reductions and treatment delays; in elderly compared with younger patients, significantly more dose reductions (9.5% vs 4.8%, p = 0.01), treatment delays (17.7% vs 9.7%, p = 0.05), particularly due to toxicity were reported (11.8% vs 7%, p = 0.05). In the FOLFIRI arm, dose reductions (4.2% vs 2.4%, p = 0.06) and treatment delays were not different. However, there was no significantly different rDI of drugs in elderly compared with younger patients, in both FOLFOXIRI and FOLFIRI arms.

Grade 3-4 diarrhea was significantly more prevalent in elderly, compared to younger patients, in both chemotherapy regimens (p = 0.005 in FOLFIRI, p = 0.017 in FOLFOXIRI arm) [32]. Moreover, diarrhea was significantly more frequent in elderly patients treated with FOLFOXIRI, compared with patients treated with FOLFIRI regimen (Table 2). No other significantly different grade 3/4 toxicities were reported, according to elderly status. No other significantly different grade 3/4 hematologic or non-hematologic toxicities were reported between young elderly (65 -75 years) and old elderly patients (>=75 years).

**Table 2:** Prevalent limiting G3-4 cumulative toxicity in young-elderly MCRC patients

	FIr-B/FOx		FOLFOXIRI		FOLFIRI	
*Number of patients*	28		52		57	
*NCI-CTC Grade*	3	4	3	4	3	4
Diarrhea (%)	21	-	30	35	14	18
Stomatitis/mucositis (%)	11	-	7	8	6	8
Asthenia (%)	11	-	7	8	5	7
Neurotoxicity (%)	-	-	8	10	-	1
Anemia (%)	-	-	3	3	1	1
Neutropenia (%)	11	-	32	38	24	28
Febrile neutropenia (%)	-	-	3	4	2	3
Thrombocytopeny (%)	-	-	1	2	1	3

#### Intensive triplet chemotherapy plus bevacizumab, FIr-B/FOx, in young-elderly

We retrospectively evaluated consecutive young-elderly patients 65 to 75 years enrolled in the previously reported phase II trial [2] and in the expanded clinical program of first-line FIr-B/FOx treatment [33], from March 2006 to November 2011: 5-FU 900 mg/m^2^, 12h-timed-flat-infusion, 2 days weekly; CPT-11 160 mg/m^2^/BEV 5 mg/kg or OXP 80 mg/m^2^, weekly alternating. Cumulative Index Rating Scale (CIRS) was used to evaluate the comorbidity status, and only patients with primary and intermediate CIRS stage were enrolled [10]. Primary CIRS stage consisted of: independent Instrumental Activity of Daily Living (IADL), and absent or mild grade comorbidities; intermediate CIRS stage consisted of dependent or independent IADL, and less than 3 mild or moderate grade comorbidities. Patients with secondary CIRS stage, consisting of more than 3 comorbidities or a severe comorbidity, with or without dependent IADL, were not enrolled. To discriminate individual safety, limiting toxicity syndromes (LTS), consisting of a LT associated or not to other limiting or G2 toxicities, were evaluated [2, 33]. LTS were classified as limiting toxicity syndromes single site (LTS-ss), characterized by the LT alone, and limiting toxicity syndromes multiple sites (LTS-ms), ≥ 2 LTs or a LT associated to other G2-3, non-limiting toxicities.

Young-elderly patients were 28 (42%) among overall MCRC patients enrolled fitting for FIr-B/FOx intensive treatment, according to inclusion criteria, WHO PS 0 89%, CIRS primary/intermediate. At a median follow-up of 17 months, ORR was 79%, liver metastasectomies 18% (37.5% in L-L patients), median PFS 11 months (3-78+), median OS 21 months (6-78+) (Table 1). Among 13 *KRAS* wild-type patients, ORR was 92%, liver metastasectomies 23% (50% in L-L patients), median PFS 14 months (4-78+ months), median OS 38 months (8-78+ months). Among 13 *KRAS* mutant patients, ORR was 77%, liver metastasectomies 15%, (20% in L-L patients), median PFS 7 months (3-69+ months), median OS 19 months (6-69+ months). Neither PFS nor OS were significantly different in *KRAS* wild-type compared with mutant patients, according to log-rank test. No *BRAF* mutations were detected.

Median rDI per cycle were 80% of the pDI for all the associated drugs. G3-4 toxicities were (Table 2): diarrhea 21%; stomatitis/mucositis 11%; asthenia 11%; neutropenia 11%. The prevalent toxicity was diarrhea, G2-G3 50%, similarly to non elderly [2]. LTS were observed in 13 out of 28 young-elderly patients (46%): LTS-ms, 11 patients (39%); LTS-ss, 2 patients (7%). LTS-ms were characterized by: LT associated to other G2-3, non-limiting toxicities, 9 patients (32%); ≥ 2 LTs, 2 patients (7%). LTS were significantly represented by LTS-ms compared to LTS-ss (chi square 3.832, p = 0.05), with respect to non elderly patients. LTS were: G2-3 diarrhea-associated, 9 patients (69.2%), 8 LTS-ms and 1 LTS-ss; G3 mucositis associated with G3 erythema, 1; G3 stomatitis/mucositis and G2 asthenia, 1; G2 neutropenia for > 2 weeks with G2 nausea, 1; G3 asthenia, 1.

#### Tailored medical treatments in patients unfit for FIr-B/FOx intensive regimen

Consecutively evaluated MCRC patients unsuitable, due to age and/or comorbidities, to be treated with triplet chemotherapy plus targeted agent, in the FIr-B/FOx expanded clinical program or to be enrolled in the ongoing phase II trial proposing intensive triplet chemotherapy plus cetuximab in *RAS* wild-type disease, were treated in clinical practice, with first line medical or surgical treatment options, chosen among those in indication for MCRC [7], and tailored according to age (< or ≥ 75 years), fitness (PS, CIRS), *RAS* genotype.

From February 2010 to October 2012, 40 patients were unfit for FIr-B/FOx intensive regimen, among 72 consecutive MCRC (56%). Patients’ distribution according to age and comorbidities was: young-elderly 22%, old-elderly 54%; CIRS stage primary 11%, intermediate 40%, secondary 42%. Medical treatment regimens were tailored according to age and comorbidity status in the individual patients. Eighteen patients (49%) were treated with triplet regimens; 15 patients (40%) with doublet regimens; 4 patients (11%) with mono regimens; 3 underwent up-front surgery.

At a median follow-up of 8 months, ORR was 37%, median PFS was 7 months (1-13+), median OS 13 months (1+-23+). Among patients treated with triplet regimens, ORR was 37.5%, median PFS 8 months (3-12), median OS 12 months (3-23+ months) (Table 3). Among patients treated with doublet regimens, ORR was 44%, median PFS 8 months (1-13+), median OS 15 months (1+-23+ months). PFS and OS were not significantly different among MCRC patients treated with triplet regimens compared to other first lines (p = 0.947 and p = 0.557, respectively), and to doublet regimens (p = 0.885 and p = 0.616, respectively). More, PFS and OS were not significantly different in non-elderly and young-elderly patients compared to old-elderly patients (p = 0.240 and p = 0.750, respectively), and in primary/intermediate compared to secondary CIRS stage patients (p = 0.494 and p = 0.364, respectively).

**Table 3:** Clinical outcome in elderly MCRC patients according to treatment selected by age and CIRS

	Medical treatment regimens		
	FIr-B/FOx	Triplet	Doublet
**Enrolled patients, No.**	28	18	15
**Elderly patients, No.**	28	12	12
**Objective Response, %**	79	37.5	44
**Median progression-free survival, months**	11	8	8
**Median overall survival, months**	21	12	15

Among 14 *KRAS* wild-type patients evaluable for activity, ORR was 50%, median PFS 8 months (1+-13+ months), median OS 13 months (1+-23+ months). Among 12 *KRAS* mutant patients evaluable for activity, ORR was 25%, median PFS 6 months (1-11 months), median OS 8 months (3-18 months). A significantly different PFS (p = 0.043), but not OS, was reported in *KRAS* wild-type compared with mutant patients. Significantly worse PFS and OS were reported in c.35 G > A *KRAS* mutant compared to wild-type (p = 0.000, and p = 0.049, respectively), and to other mutant patients (p = 0.020, and p = 0.048, respectively).

## DISCUSSION

Fit young elderly patients, PS<2, treated with triplet regimens consisting of chemotherapeutic drugs, or BEV addiction to doublets, or doublets plus EGFR-inhibitors in *KRAS* wild-type patients, demonstrated clinical outcome and safety profile equivalent to younger patients [3, 5, 8, 1]. More intensive FIr-B/FOx regimen [2, 9, 34] obtained ORR 79%, median PFS 11, median OS 21 months in fit young-elderly patients. FIr-B/FOx was feasible at median rDI 80%. Prevalent G3-4 toxicities were diarrhea (21%), stomatitis/mucositis (11%), asthenia (11%), neutropenia (11%). Good safety profile, particularly regarding haematological toxicity, could be related to weekly alternating schedule of triplet chemotherapy regimen added to bevacizumab. Individual LTS were reported in 46% young-elderly patients, mainly including diarrhea (69.2%), and significantly more represented by LTS-ms compared to LTS-ss (chi square 3.832, p = 0.05), with respect to non elderly patients. Our retrospective exploratory analysis, evaluated in a small cohort of MCRC patients that requires further prospective validation, showed that intensive FIr-B/FOx schedule is equivalently safe and feasible, without severe adverse events related to BEV, in young-elderly patients, selected by favourable PS, functional and comorbidity status, with LTS-ms significantly increased compared to LTS-ss, compared to non-elderly patients. Young-elderly MCRC patients suitable for intensive FIr-B/FOx regimen should be carefully selected based on comorbidity and functional status, and monitored for individual safety in clinical practice (Table 4). Elderly MCRC patients are prevalent and first line medical treatment should be selected according to a decision-making process integrating the evaluation of patient's fitness for intensive medical treatments with reported increasing effectiveness and toxicity.

**Table 4:** Selection and monitoring of elderly MCRC patients suitable for intensive medical treatment in clinical practice

Parameters	Intensive medical treatment	Not intensive medical treatment
Age	< 75 years	≥ 75 years
CIRS	Primary/intermediate	Secondary
Individual LTS	Absent	Present (prevalently LTS-ms)

Abbreviations: CIRS, Cumulative Illness Rating Scale; LTS, limiting toxicity syndromes; LTS-ms, LTS multiple sites.

Retrospective analysis of randomized clinical trials showed that doublets CPT-11, or OXP, added to fluoropyrimidin in older patients eligible for clinical study reported ORR 18-59.4%, PFS 4.9-10.0 months and OS 8.5-20.7 months [15–22, 32, 30]. In elderly patients, significantly increased PFS up to 9.2-9.3 and OS up to 17.4-19.3 months, were reported with BEV addition to 5-FU-based chemotherapy [24, 25]. Triplet chemotherapeutic drugs or BEV added to doublets reached ORR 34.9-45.9%, PFS 7.9-9.3 months and OS 17.4-20.5 months [25–27]. The positive benefits in terms of efficacy and tolerability highlighted by these trials represented the main reason that led the international scientific societies (NCCN and ESMO) to recommend chemotherapy for elderly patients deemed fit for standard chemotherapy [45, 46]. In particular, our tailored clinical approach, characterized by the evaluation of elderly status and/or CIRS (Table 4), and prevalently tailoring doublets and triplets in MCRC patients unfit for intensive first line FIr-B/FOx, reported ORR 37%, PFS 7 months and OS 13 months. PFS and OS were not significantly different according to administered treatment regimens, triplet versus doublet, elderly status, or CIRS stage. In the FOCUS2 trial, evaluating first line OXP addiction to 80% dose 5-FU or capecitabine in old-elderly and/or frail MCRC patients, ORR was significantly improved up to 35%, with PFS 5.8 months [22]. PS was reported as significantly related to clinical outcome, regardless of treatment: ORR 43.8% vs 32%, PFS 7.6 vs 4.9 months, OS 17.3 and 8.5 months, in PS 1 compared to PS 2 patients, respectively [22]. In the HORG-FOLFOXIRI trial, no different clinical outcome was observed in elderly versus non-elderly patients; significantly lower clinical outcome was reported in patients with PS 2 [31, 32]. Liver metastasectomies were reported in 1.3% and 4.4% patients in FOLFIRI and FOLFOXIRI arms, respectively [32] and can achieve OS 43 months, not significantly different from younger patients in the experience of liver resection in elderly patients [35]. Morbidity and/or mortality after liver surgery were significantly higher in elderly patients (8%) [36].

Published studies showed that limiting toxicities were not significantly different in elderly patients treated with 5-FU or CPT-11 [14–16], slightly increased with FOLFOX [21], significantly increased by capecitabine (40%), while not by the addition of OXP [22]. Limiting diarrhoea was significantly higher with FOLFIRI and FOLFOXIRI [29, 30]. PS 2 was significantly associated with increased grade 3/4 neutropenia, febrile neutropenia, diarrhoea, fatigue, compared with PS 0-1 [30–32]. In elderly patients, BEV addition to chemotherapy was significantly associated with increased arterial thromboembolism [37], while not to other adverse events [24–27].

In clinical practice, selection of patients eligible for intensive medical treatment and to achieve optimal activity and clinical outcome could be performed by the evaluation of age, PS, CIRS and careful monitoring of individual safety using LTS (Table 4). Patients unfit for first line intensive FIr-B/FOx, due to age (≥ 75 years) and/or comorbidity status, were prevalent (56%), mostly elderly (76%), specifically old-elderly (54%), prevalently PS 1-2 (59%), intermediate/secondary CIRS stage (89%), O/MM disease (79%) [7]. Patients unfit for FIr-B/FOx showed worse PFS and OS. No significantly higher morbidity, nor mortality rates were showed in unfit patients who underwent secondary resection of liver metastases, reported as significantly more frequent in elderly patients (8%) [36]. In patients unfit for FIr-B/FOx, a significantly different PFS, while not OS, was reported in *KRAS* wild-type compared to mutant patients. More, significantly worse clinical outcome (PFS and OS) may be influenced by *KRAS* c.35 G > A mutant genotype, compared to wild-type and/or other mutant, confirming that *KRAS* genotype, and specifically c.35 G > A mutant, confers different biological aggressiveness [38–41], less effectively overcome by triplet and doublet medical treatment regimens conventionally administered in clinical practice. A careful decision-making process including CIRS and monitoring of individual LTS can be used to properly select first line intensive FIr-B/FOx or tailored medical treatment in young-elderly patients. In unfit MCRC patients, *KRAS* genotype may significantly explain different PFS, and c.35 G > A *KRAS* mutant a significantly worse PFS and OS, compared to wild-type and other mutant.

## MATERIALS AND METHODS

We revised intensive medical treatment consisting of triplet chemotherapy regimens or more intensive triplet chemotherapy plus anti-angiogenic drug, bevacizumab, in elderly MCRC patients, that we previously developed, discussed compared with tailored medical regimens selected for unfit MCRC in clinical practice [2, 7, 32], and discussed in the scenario of therapies proposed for elderly patients. This review comprehensively evaluate activity and clinical outcome of first-line medical treatment of elderly MCRC patients to propose a careful decision-making process including age, performance status, and Cumulative Index Rating Scale (CIRS) and monitoring of individual limiting toxicity syndromes (LTS), to properly select first line intensive or tailored medical treatment in young-elderly and elderly patients [7, 8, 2, 9], and aim to underline the prognostic relevance of *KRAS* genotype, and specifically the prevalent *KRAS* c.35 G > A mutant status, that can discriminate significantly different clinical outcome particularly in unfit MCRC patients.

## References

[1] Schmoll HJ, Van Cutsem E, Stein A, Valentini V, Glimelius B, Haustermans K, Nordlinger B, van de Velde CJ, Balmana J, Regula J, Nagtegaal ID, Beets-Tan RG, Arnold D (2012). ESMO Consensus Guidelines for management of patients with colon and rectal cancer. A personalized approach to clinical decision making. Annals of Oncology.

[2] Bruera G, Santomaggio A, Cannita K, Baldi PL, Tudini M, De Galitiis F, Mancini M, Marchetti P, Antonucci A, Ficorella C, Ricevuto E (2010). association of weekly alternating 5-Fluorouracil, Irinotecan, Bevacizumab and Oxaliplatin (FIr-B/FOx) in first line treatment of metastatic colorectal cancer: a phase II study. BMC Cancer.

[3] Bruera G, Ricevuto E (2010). Intensive chemotherapy of metastatic colorectal cancer: weighing between safety and clinical efficacy. Evaluation of Masi G, Loupakis F, Salvatore L, et al. Bevacizumab with FOLFOXIRI (irinotecan, oxaliplatin, fluorouracil, and folinate) as first-line treatment for metastatic colorectal cancer: a phase 2 trial. Lancet Oncol.

[4] Bruera G, Cannita K, Giuliante F, Lanfiuti Baldi P, Vicentini R, Marchetti P, Nuzzo G, Antonucci A, Ficorella C, Ricevuto E (2012). Effectiveness of liver metastasectomies in patients with metastatic colorectal cancer treated with FIr-B/FOx triplet chemotherapy plus bevacizumab. Clin Colorectal Cancer.

[5] Ficorella C, Bruera G, Cannita K, Porzio G, Baldi PL, Tinari N, Natoli C, Ricevuto E (2012). Triplet chemotherapy in patients with metastatic colorectal cancer: toward the best way to safely administer a highly active regimen in clinical practice. Clin Colorectal Cancer.

[6] Bruera G, Cannita K, Di Giacomo D, Lamy A, Troncone G, Dal Mas A, Coletti G, Frébourg T, Sabourin JC, Tosi M, Ficorella C, Ricevuto E (2012). Prognostic value of KRAS genotype in metastatic colorectal cancer (MCRC) patients treated with intensive triplet chemotherapy plus bevacizumab (FIr-B/FOx) according to extension of metastatic disease. BMC Medicine.

[7] Bruera G, Cannit K, Giordano AV, Vicentini R, Ficorell C, Ricevuto E (2014). Prognostic relevance of KRAS genotype in metastatic colorectal cancer patients unfit for FIr-B/FOx intensive regimen. Int J Oncol.

[8] Masi G, Vasile E, Loupakis F, Cupini S, Fornaio L, Baldi G, Salvatore L, Cremolini C, Stasi I, Brunetti I, Fabbri MA, Pugliesi M, Trenta P (2011). Randomized Trial of Two Induction Chemotherapy Regimens in Metastatic Colorectal Cancer: An Updated Analysis. J Natl Cancer Inst.

[9] Loupakis F, Cremolini C, Masi G, Lonardi S, Zagonel V, Salvatore L, Cortesi E, Tomasello G, Ronzoni M, Spadi R, Zaniboni A, Tonini G, Buonadonna A (2014). Initial therapy with FOLFOXIRI and bevacizumab for metastatic colorectal cancer. N Engl J Med.

[10] Extermann M, Overcash J, Lyman GH, Parr J, Balducci L (1998). Comorbidity and functional status are independent in older cancer patients. J Clin Oncol.

[11] Pallis AG, Papamichael D, Audisio R, Peeters M, Folprecht G, Lacombe D, Van Cutsem E (2010). EORTC Elderly Task Force experts’ opinion for the treatment of colon cancer in older patients. Cancer Treatment Reviews.

[12] Papamichael D, Audisio R, Horiot JC, Glimelius B, Sastre J, Mitry E, Van Cutsem E, Gosney M, Kohne CH, Aapro M (2009). Treatment of the elderly colorectal cancer patient: SIOG expert recommendations. Annals of Oncology.

[13] Audisio RA, Papamichael D (2012). Treatment of colorectal cancer in older patients. Nat Rev Gastroenterol Hepatol.

[14] Folprecht G, Cunningham D, Ross P, Glimelius B, Di Costanzo F, Wils J, Scheithauer W, Rougier P, Aranda E, Hecker H, Kohne CH (2004). Efficacy of 5-fluorouracil-based chemotherapy in elderly patients with metastatic colorectal cancer: a pooled analysis of clinical trials. Ann Oncol.

[15] Popescu RA, Norman A, Ross PJ, Parikh B, Cunningham D (1999). Adjuvant or palliative chemotherapy for colorectal cancer in patients 70 years or older. J Clin Oncol.

[16] Chiara S, Nobile MT, Vincenti M, Lionetto R, Gozz A, Barzacchi MC, Sanguineti O, Repetto L, Rosso R (1998). Advanced colorectal cancer in the elderly: results of consecutive trials with 5-fluorouracil-based chemotherapy. Cancer Chemother Pharmacol.

[17] Folprecht G, Seymour MT, Saltz L, Douillard JY, Hecker H, Stephens RJ, Maughan TS, Van Cutsem E, Rougier P, Mitry E, Schubert U, Kohne CH (2008). Irinotecan/Fluorouracil combination in first-line therapy of older and younger patients with metastatic colorectal cancer: combined analysis of 2,691 patients in randomized controlled trials. J Clin Oncol.

[18] Mitry E, Douillard JY, Van Cutsem E, Cunningham D, Magherini E, Mery-Mignard D, Awad L, Rouigier P (2004). Predictive factors of survival in patients with advanced colorectal cancer: an individual data analysis of 602 patients included in irinotecan phase III trials. Ann Oncol.

[19] Goldberg RM, Tabah-Fisch I, Bleiberg H, de Gramont A, Tournigand C, Andre T, Rothenberg ML, Green E, Sargent DJ (2006). Pooled analysis of safety and efficacy of oxaliplatin plus fluorouracil/leucovorin administered bimonthly in elderly patients with colorectal cancer. J Clin Oncol.

[20] Figer A, Perez-Staub N, Carola E, Tournigand C, Lledo G, Flesch M, Barcelo R, Cervantes A, André T, Colin P, Louvet C, de Gramont A (2007). FOLFOX in patients aged between 76 and 80 years with metastatic colorectal cancer an exploratory cohort of the OPTIMOX1 study. Cancer.

[21] Seymour MT, Thompson LC, Wasan HS, Middleton G, Brewster AE, Shepherd SF, O’Mahony MS, Maughan TS, Parmar M, Langley RE (2011). Chemotherapy options in elderly and frail patients with metastatic colorectal cancer (MRC FOCUS2): an open-label, randomised factorial trial. Lancet.

[22] Hurwitz HI, Fehrenbacher L, Novotny WF, Cartwright T, Hainsworth J, Meropol NJ, Heim W, Berlin J, Baron A, Griffing S, Holmgren E, Ferrara N, Fyfe G (2004). Bevacizumab plus irinotecan, fluorouracil, and leucovorin for metastatic colorectal cancer. N Engl J Med.

[23] Kabbinavar FF, Hurwitz HI, Yi J, Sarkar S, Rosen O (2008). Addition of bevacizumab to fluorouracil-based first-line treatment of metastatic colorectal cancer: pooled analysis of cohorts of older patients from two randomized clinical trials. J Clin Oncol.

[24] Cassidy J, Saltz LB, Giantonio BJ, Kabbinavar FF, Hurwitz HI, Roh UP (2010). Effect of bevacizumab in older patients with metastatic colorectal cancer: pooled analysis of four randomized studies. J Cancer Res Clin Oncol.

[25] Kozloff MF, Berlin J, Flynn PJ, Kabbinavar F, Ashby M, Don W, Sing AP, Grothey A (2010). Clinical outcomes in elderly patients with metastatic colorectal cancer receiving bevacizumab and chemotherapy: results from the BRiTE observational cohort study. Oncology.

[26] Van Cutsem E, Rivera F, Berry S, Kretzschmar A, Michael M, Di Bartolomeo M, Mazier MA, Canon JL, Georgoulias V, Peeters M, Bridgewater J, Cunningham D (2009). Safety and efficacy of first-line bevacizumab with FOLFOX, XELOX, FOLFIRI and fluoropyrimidines in metastatic colorectal cancer: the BEAT study. Annals of Oncology.

[27] Meyerhardt JA, Jackson McCleary N, Niedzwiecki D, Hollis D, Venook A, Mayer R, Goldberg R (2009). Impact of age and comorbidities on treatment effect, tolerance, and toxicity in metastatic colorectal cancer (mCRC) patients treated on CALGB 80203. J Clin Oncol.

[28] Douillard J, Siena S, Cassidy J, Tabernero J, Burkes R, Barugel M, Humblet Y, Bodoky G, Cunningham D, Jassem J, Rivera F, Kocàkova I, Ruff P (2010). Randomized, phase III trial of Panitumumab with infusional fluorouracil, leicovorin, and oxaliplatin (FOLFOX4) versus FOLFOX4 alone as first-line treatment in patients with previously untreated metastatic colorectal cancer: the PRIME trial. J Clin Oncol.

[29] Sargent DJ, Kohne CH, Sanoff HK, Bot BM, Seymour MT, de Gramont A, Porschen R, Saltz LB, Rougier P, Tournigand C, Douillard JY, Stephens RJ, Grothey A, Goldberg RM (2009). Pooled safety and efficacy analysis examining the effect of performance status on outcomes in nine first-line treatment trials using individual data from patients with metastatic colorectal cancer. J Clin Oncol.

[30] Souglakos J, Andrulakis N, Syrigos K, Polyzos A, Ziras N, Athanasiadis A, Kalolyris S, Tsousis S, Kouroussis CH, Vamvakas L, Kalykaki A, Samonis G, Mavroudis D, Georgoulias V (2006). FOLFOXIRI (folin acid, 5-fluorouracil, oxaliplatin and irinotecan) vs FOLFIRI (folin acid, 5-fluorouracil and irinotecan) as first-line treatment in metestatic colorectal cancer (MCC): a multicentre randomised phase III trial from the Hellenic Oncology Research Group (HORG). British Journal of Cancer.

[31] Vamvakas L, Athanasiadis A, Karampeazis A, Kakolyris S, Polyzos A, Kouroussis C, Ziras N, Kalbakis K, Georgoulias V, Souglakos J (2010). Clinical outcome of elderly patients with metastatic colorectal cancer treated with FOLFOXIRI versus FOLFIRI: Subgroup analysis of a randomized phase III trial from the Hellenic Oncology Research Group (HORG). Crit Rev Oncol Hematol.

[32] Bruera G, Cannita K, Giordano AV, Vicentini R, Ficorella C, Ricevuto E (2013). Effectiveness and safety of intensive triplet chemotherapy plus bevacizumab, FIr-B/FOx, in young-elderly Metastatic Colorectal Cancer (MCRC) patients. BioMed Res Int.

[33] Garufi C, Torsello A, Tumolo S, Ettorre GM, Zeuli M, Campanella C, Vennarecci G, Mottolese M, Sperduti I, Cognetti F (2010). Cetuximab plus chronomodulated irinotecan, 5-fluorouracil, leucovorin and oxaliplatin as neoadiuvant chemotherapy in colorectal liver metastases: POCHER trial. Br J Cancer.

[34] Adam R, Frilling A, Elias D, Laurent C, Ramos E, Capussotti L, Poston GJ, Wicherts DA, de Haas J, the LiverMetSurvey Centres (2010). Liver resection of colorectal metastases in elderly patients. British Journal of Surgery.

[35] Figueras J, Ramos E, López-Ben S, Torras J, Albiol M, Llado L, González HD, Rafecas A (2007). Surgical treatment of liver metastases from colorectal carcinoma in elderly patients. When is it worthwhile?. Clin Transl Oncol.

[36] Scappaticci FA, Skillings JR, Holden SN, Gerber HP, Miller K, Kabbinavar F, Bergsland E, Ngai J, Holmgren E, Wang J, Hurwitz H (2007). Arterial thromboembolic events in patients with metastatic carcinoma treated with chemotherapy and bevacizumab. J Natl Cancer Inst.

[37] Bruera G, Cannita K, Di Giacomo D, Lamy A, Frébourg T, Sabourin JC, Tosi M, Ficorella C, Ricevuto E (2013). Worse prognosis of KRAS c.35 G > A mutant metastatic colorectal cancer (MCRC) patients treated with intensive triplet chemotherapy plus bevacizumab (FIr-B/FOx). BMC Medicine.

[38] Bruera G, Cannita K, Giordano AV, Vicentini R, Ficorella C, Ricevuto E (2014). Differential prognosis of metastatic colorectal cancer patients post-progression to first line triplet chemotherapy plus bevacizumab, FIr-B/FOx, according to second line treatment and KRAS genotype. Int J Oncol.

[39] Bruera G, Cannita K, Tessitore A, Russo A, Alesse E, Ficorella C, Ricevuto E (2015). The prevalent KRAS exon 2 c.35 G > A mutation in metastatic colorectal cancer patients: a biomarker of worse prognosis and potential benefit of bevacizumab-containing intensive regimens?. Crit Rev Oncol Hematol.

[40] Guerrero S, Casanova I, Farrè L, Mazo A, Capellà G, Mangues R (2000). K-ras codon 12 mutation induces higher level of resistance to apoptosis and predisposition to anchorage-independent growth than codon 13 mutation or proto-oncogene overexpression. Cancer Res.

[41] Vincenzi B, Cremolini C, Sartore-Bianchi A, Russo A, Mannavola F, Perrone G, Pantano F, Loupakis F, Rossini D, Ongaro E, Bonazzina E, Dell’Aquila E, Imperatori M (2015). Prognostic significance of K-Ras mutation rate in metastatic colorectal cancer patients. Oncotarget.

[42] Bronte G, Silvestris N, Castiglia M, Galvano A, Passiglia F, Sortino G, Cicero G, Rolfo C, Peeters M, Bazan V, Fanale D, Giordano A, Russo A (2015). New findings on primary and acquired resistance to anti-EGFR therapy in metastatic colorectal cancer: do all roads lead to RAS?. Oncotarget.

[43] Vamvakas L, Matikas A, Karampeazis A, Hatzidaki D, Kakolyris S, Christophylakis C, Boukovinas I, Polyzos A, Georgoulias V, Souglakos J (2014). Capecitabine in combination with oxaliplatin and bevacizumab (AXELOX) as 1st line treatment for fit and vulnerable elderly patients (aged >70 years) with metastatic colorectal cancer (mCRC): a multicenter phase II study of the Hellenic Oncology Research Group (HORG). BMC Cancer.

[44] Feliu J, Salud A, Safont MJ, García-Girón C, Aparicio J, Vera R, Serra O, Casado E, Jorge M, Escudero P, Bosch C, Bohn U, Pérez-Carrión R (2014). First-line bevacizumab and capecitabine-oxaliplatin in elderly patients with mCRC: GEMCAD phase II BECOX study. Br J Cancer.

[45] https://www.nccn.org/store/login/login.aspx?ReturnURL=https://www.nccn.org/professionals/physician_gls/pdf/colon.pdf

[46] Van Cutsem E, Cervantes A, Adam R, Sobrero A, Van Krieken JH, Aderka D, Aranda Aguilar E, Bardelli A, Benson A, Bodoky G, Ciardiello F, D’Hoore A, Diaz-Rubio E ESMO consensus guidelines for the management of patients with metastatic colorectal cancer. Ann Oncol.

